# Efficacy and Tolerability of Gabapentin in Adults with Sleep Disturbance in Medical Illness: A Systematic Review and Meta-analysis

**DOI:** 10.3389/fneur.2017.00316

**Published:** 2017-07-14

**Authors:** Guang Jian Liu, Md Rezaul Karim, Li Li Xu, Song Lin Wang, Chao Yang, Li Ding, Yun-Fu Wang

**Affiliations:** ^1^Department of Neurology, Taihe Hospital, Hubei University of Medicine, Shiyan, China; ^2^Department of Neurology, Wuhan Dongxihu District People’s Hospital, Wuhan, China

**Keywords:** sleep disturbance, gabapentin, efficacy, tolerability, meta-analysis

## Abstract

**Background and purpose:**

The aim of this study was to systematically review the efficacy and tolerability of gabapentin in the treatment of sleep disturbance in patients with medical illness.

**Methods:**

PubMed was searched for randomized, double-blinded, placebo-controlled trials that reported sleep changes during gabapentin treatment up to November 2015.

**Findings:**

This review included 26 studies involving 4,684 participants. Except for Composite Endpoint 3 [standardized mean difference (SMD) = 0.09, 95% confidence interval (CI): −0.05–0.22] compared with the placebo group, the gabapentin group showed superior outcomes on our endpoints: Composite Endpoint 1 (SMD = 0.50, 95% CI: 0.28–0.71), Composite Endpoint 2 (SMD = −0.53, 95% CI: −0.77 to −0.30), Composite Endpoint 4 (SMD = −0.38, 95% CI: −0.58 to −0.19), Composite Endpoint 5 [risk ratio (RR) = 1.79, 95% CI: 1.24–2.58], and Composite Endpoint 6 (RR = 0.48, 95% CI: 0.32–0.72). However, the patients in the gabapentin group showed worse tolerance than those in the placebo group (RR = 1.38, 95% CI: 1.08–1.76).

**Implications:**

This study is the first to systematically assess the clinical value of gabapentin for the treatment of sleep disorders. We found that regardless the type of sleep outcomes, gabapentin displayed stable treatment efficacy for sleep disturbance in patients with medical illness. However, when an average dose of approximately 1,800 mg/day was used, the risk of treatment discontinuation or drug withdrawal was relatively high. We recommend that further studies confirm these findings in patients with primary sleep disorders.

## Introduction

Sleep disorders have been always a disturbing public health issue, not only because they affect quality of life, increase the patient’s risk of cardio-cerebrovascular disease (1, 2) and death (2, 3), weaken social productivity, and increase medical burdens (4, 5) but also because unlike other diseases with a phase-wise pattern, they cannot be cured using multiphase treatment. Although phenobarbital, benzodiazepine hypnotics, Z-drugs, antidepressants, and melatonin receptor agonists can all contribute to a certain extent (6, 7), few of these treatments can either restore patients’ normal sleep structure or completely cure sleep disorders.

Gabapentin, an apha-2-delta voltage-gated calcium channel ligand (8) that is widely used for the treatment of epilepsy, neuropathic pain, and restless legs syndrome, can enhance slow-wave sleep in both normal individuals (9) and epileptic patients (10, 11) and can improve slow-wave sleep and sleep efficiency and reduce nighttime awakening in patients with primary sleep disorders (12). However, these findings have not been verified with randomized controlled trials. Clinical studies have revealed that gabapentin could improve the objective and subjective outcomes of sleep disturbance in patient with medical illness (13–37). Gabapentin Enacarbil (GEn) or XP13512 is a prodrug of gabapentin, used as an anticonvulsant and for pain relief in postherpetic neuralgia. This new formulation of gabapentin was designed for increased oral bioavailability over gabapentin. It provides reliable drug absorption and consistent bioavailability (16). Nevertheless, the results derived from these studies had certain inconsistencies and did not undergo any systematical evaluation. Through a systematic review of the use of gabapentin to treat restless legs syndrome, neuropathic pain, alcohol dependence, hot flashes in menopause, fibromyalgia, phantom limb pain, human immunodeficiency virus (HIV)-associated sensory neuropathies, and bipolar disorder, this study attempted to evaluate the efficacy and tolerability of gabapentin for the treatment of sleep disturbance in patients with medical illness.

## Materials and Methods

This systematic review and meta-analysis were performed according to the Preferred Reporting Items for Systematic Reviews and Meta-Analyses statement (PRISMA) (38). There are no ethical issues involved in our study because our data were based on published studies.

### Data Sources and Search

PubMed was searched for all clinical trials related to the present research topic (up to November 8, 2015). The keywords selected from the Medical Subject Headings (MeSH) included intervention, study type, and endpoint event. The search range was “title/abstract/keywords.” No language restrictions were applied. In addition, we screened the reference lists of all included trials to identify additional eligible studies. Detailed information regarding the search terms used in the literature search is provided in the Supplementary Material.

### Study Selection

#### Eligible Trials

(1) Participants: all included patients were 18 years or older and had/did not have a record of baseline sleep status; (2) intervention: the patients in the treatment group received gabapentin, gabapentin enacarbil, or XP13512 (Gabapentin), and the patients in the control group received placebos with a treatment duration of at least 7 days; (3) endpoints: all included trials reported sleep changes and treatment discontinuation or drug withdrawal events that were possibly or probably related to the study drugs; (4) study type: randomized, double-blinded, controlled trials were included.

### Data Extraction

Using a unified form, two investigators independently extracted the data and created the data spreadsheet, which were then cross-checked to ensure data accuracy. Disagreements were resolved by consensus. The extracted data mainly included the six composite endpoints and treatment discontinuation or drug withdrawal events that were possibly or probably related to the study drugs.

### Endpoint Definitions

Because of the diversity of outcomes reported in the included trials, only a limited number of trials provided data that could be pooled for each meta-analysis. To reach a sufficient statistical level, we introduced the concept of “composite endpoint” to pool the data related to sleep outcomes with similar significance and a consistent direction.

Based on the treatment outcomes and relevant data provided by the original trials, seven composite endpoints were analyzed for evaluation. Composite Endpoints 1–6 were used to evaluate the efficacy of gabapentin, and Composite Endpoint 7 was used to evaluate treatment discontinuation or drug withdrawal events that were possibly or probably associated with gabapentin. Composite Endpoints 1–4 indicated sleep improvement after treatment. Specifically, Composite Endpoint 1 represented the net increase in the evaluation indices provided in the trials in which the index values increased, but the baseline values were not provided. Composite Endpoint 2 represented the net decrease of evaluation indices provided in the trials in which the index values decreased but the baseline values were not provided. Composite Endpoint 3 and Composite Endpoint 4 represented the posttreatment values of the evaluation indices provided in the trials in which the index values increased and the trials in which the index values decreased (none of these trials provided the baseline values), respectively. Composite Endpoint 5 (Excellent, 0 or Good) represented the sleep outcomes that received the highest grades in the survey, e.g., the overall quality of sleep was evaluated as “Excellent,” or the ability to function was evaluated as “Good,” or the number of nighttime awakenings caused by RLS symptoms was 0, or the number of hours awake per night because of RLS symptoms was 0 in the past week. Composite Endpoint 6 (Poor, ≥3, ≥5, or 7) represented the sleep outcomes that were graded the lowest in the survey, e.g., the overall quality of sleep was evaluated as “Poor,” or the ability to function was evaluated as “Poor,” or the number of nighttime awakenings caused by RLS symptoms was ≥5, or the number of hours awake per night because of RLS symptoms was ≥3, or the number of nights with RLS symptoms was 7 in the past week.

### Quality Assessment

Two investigators evaluated the methodological quality of all included trials according to the Cochrane Collaboration’s tool for assessing bias [the Reviewer’s Handbook (39)].

### Grading of Recommendations Assessment, Development, and Evaluation (GRADE) Classification

Based on the GRADE study group criteria (20), we graded the evidence quality for all of the endpoints.

### Data Synthesis and Analysis

Based on the formula and endpoint definition, the values of the same endpoints in each trial were pooled first and then the data from different trials were pooled together for analysis. The standardized mean difference (SMD) and risk ratio (RR) were used to assess the abovementioned endpoints. Prior to the meta-analysis of each endpoint, statistical heterogeneity across the various trials was tested using Chi-square test. A *P*-value greater than the nominal level of 0.10 and *I*^2^ ≤40% indicated a lack of heterogeneity across trials, allowing for the use of a fixed-effects model; otherwise, a random-effects model was used. The inverse variance method was used for continuous variables, and the Mantel–Haenszel method was used for dichotomous variables. In addition, a sensitivity analysis was conducted by removing each trial one at a time, and the publication bias was evaluated using the Egger test.

SPSS Predictive Analytics Software version 18.0 (SPSS, Inc., Chicago, IL, USA) was used for the Chi-square tests, and Stata Statistical Software version SE 12.0 (Stata Corp. LP, College Station, TX, USA) was used for all other analyses.

## Results

### Search Results and Trial Characteristics

Ninety-eight records were identified through database searches and were screened by reading titles, abstracts, and part of main text. After irrelevant papers, observational studies, duplicates, and trials that used non-placebo control drugs were excluded, 26 papers (13–19, 21–37, 40, 41) met the inclusion criteria. The included publications comprised eight RLS-related trials, eight neuropathic pain-related trials, and three alcohol dependence-related trials, two trials involving hot flashes in menopause, one fibromyalgia-related trial, one trial involving phantom limb pain, one trial involving HIV-associated sensory neuropathies, and one bipolar disorder-related trial. Among the included studies, six trials were included only for systematic review and 20 trials were included for meta-analysis.

**Figure 1:**
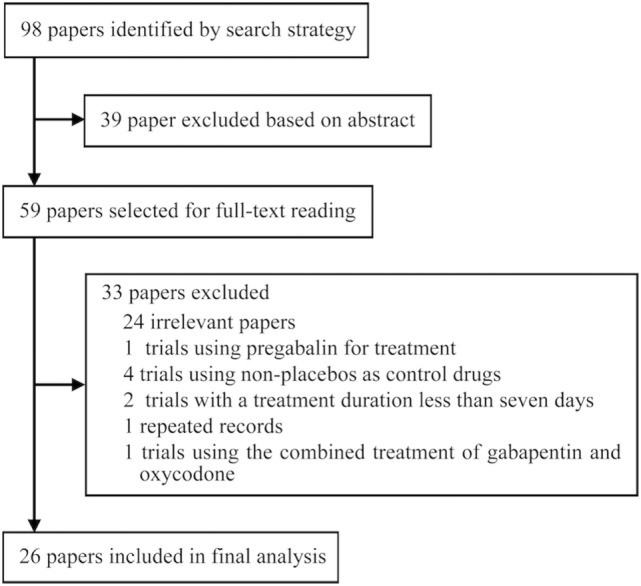
Flow diagram of the screening process.

The included 26 trials involved 4,684 patients. The average follow-up length was 11.07 weeks/per patient, and the total follow-up time was 997.23 patient-years. The average age of 83.50% of the patients was 55.45 (±13.45) years. Among 96.50% of patients, males accounted for 42.73%; among 90.67% of patients, the average length of disease course was at least 6.23 (±9.76) years. The initial dose of gabapentin was 300 or 600 mg/day; after the dose-increasing phase, the minimum dose was 600 mg/day and the maximum dose was 3,600 mg/day, with an average dose of 1,793.92 mg/day. Figure 1 presents the screening process used in the study, Table 1 lists the main characteristics of all included trials.

**Table 1:** Characteristics of the included studies.

Reference	Participants					Intervention		Sleep outcome	Study design and treatment duration (weeks)
	Diagnoses	Sample size	Age (years)^a^	Male (%)	Illness duration (years)^a^	Gabapentin group	Control group		
Anton et al. (13)	Alcohol dependence	100	44.82 ± 9.53	82.45	NR	Gabapentin combined with naltrexone (50 mg/day); gabapentin: the initial dose was 300 mg prior to bedtime, increased to 1,200 mg/day at night from the fifth day	Identical placebo	Insomnia sleep index (42), Epworth Sleepiness Scale (43); adverse effects	RCT; 6
Brower et al. (41)	Alcohol dependence	21	46 (30.8–60)^b^/44 (41–54)^b^	52.38	NR	Gabapentin: the initial oral dose was 300 mg 45 min before bedtime, increased to 1,500 mg/day at bedtime within 10 days	Identical placebo	Sleep problems questionnaire; Sleep diaries; Polysomnography parameters; adverse effects	RCT; 6
Hahn et al. (21)	Human immunodeficiency virus-associated sensory neuropathies	25	46 (27–59)^b^/44 (35–61)^b^	80	0.76 (median)	Gabapentin: the dose was adjusted every 4 days until it reached 1,200 mg/day after more than 2 weeks	Matching placebo	Mean sleep interference score^c^; adverse effects	RCT; 4
Rowbotham et al. (30)	Postherpetic neuralgia	229	73 (40–90)^b^/74 (39–89)^b^	52.44	2.39 (median)	Gabapentin: the initial dose was 300 mg, increased to 3,600 mg/day at night from the fourth week	Identical placebo	Mean sleep interference score^c^; adverse effects	RCT; 8
Rice et al. (29)	Postherpetic neuralgia	334	76.3 (36.1–90.8)^d^/74.9 (28.9–94.8)^d^	41.32	2.19 (median)	Gabapentin: the initial dose was 300 mg/day, increased to 1,800 or 2,400 mg/day within 2 weeks	Identical placebo	Mean sleep interference score^c^; adverse effects	RCT; 7
Garcia-Borreguero et al. (18)	RLS	44	NR	NR	NR	Gabapentin: the dose was initially 600 mg/day and was adjusted every 2 weeks to a maximum dose of 2,400 mg/day (1200 hours and 2000 hours)	Identical placebo	Pittsburgh sleep quality index global score (44); polysomnography parameters; adverse effects	RCT; 6 (excluding the washout period and crossover period)
Gordh et al. (19)	Neuropathic pain	120	NR	NR	≥0.5	Gabapentin: the initial dose was 300 mg/day, increased to 2,400 mg/day from the third week	Identical placebo	Mean sleep interference score^e^; adverse effects	RCT; 5 (excluding the washout period and crossover period)
Lal et al. (25)	RLS	217	48.0 ± 12.70	64.06	13.39 ± 13.68	Gabapentin enacarbil: the initial dose was 600 mg/day, increased to 1,200, 1,800, or 2,400 mg/day within 9 days	Identical placebo	Post-sleep questionnaire; tolerability assessments	RCT; 12
Mason et al. (27)	Alcohol dependence	150	44.53 ± 11.01	56.67	14.43 ± 9.85	Gabapentin: the initial dose was 300 mg/day, increased to 900 or 1,800 mg/day within 6 days	Identical placebo	Pittsburgh sleep quality index global score (44); adverse effects	RCT; 12
Bone et al. (40)	Phantom limb pain	19	56.25 ± 17.5	78.95	1.5 (median)	The first phase was gabapentin treatment (12 weeks): the initial dose was 300 mg/day, gradually increased to 2,400 mg/day; the second phase was placebo treatment (6 weeks), with 1 week of washout between the two phases	6 weeks of placebo treatment and 12 weeks of gabapentin treatment	Mean sleep interference score^c^; adverse effects	RCT plus crossover; 18
Backonja et al. (15)	Diabetic neuralgia	165	53 ± 10.32	60	11.61 ± 9.15	Gabapentin: 900 mg/day for the first week, increased to 3,600 mg/day from the fourth week	Identical placebo	Mean sleep interference score^c^; adverse effect	RCT; 8
Arnold et al. (14)	Fibromyalgia	150	48.25 ± 11.22	90	≥0.5	Gabapentin: 300 mg before bedtime at the first week, increased to 600 mg twice a day plus 1,200 mg before bedtime from the sixth week	Identical placebo	Medical outcomes study sleep problems index score (45); adverse effects	RCT; 12
Winkelman et al. (36)	RLS	272	52.0 ± 12.7	41.98	NR	Gabapentin enacarbil: 600 mg/day initially, increased to 1,200 mg/day from the fourth day to the end of the first 4 weeks, followed by another 4-week placebo treatment phase after a 7-day dose-decreasing period and a 7-day washout period	4 weeks of identical placebo, followed by 4 weeks of gabapentin enacarbil treatment after 2 weeks of washout	Polysomnography parameters; subjective post-sleep diary; tolerability assessments	RCT plus crossover; 8
Vieta et al. (33)	Bipolar disorder	25	46.87 ± 14.74	28	18.79 ± 10.90	Gabapentin: 1,200 mg/day initially, adjusted to 900 mg/day within 1 week according to the symptoms and patient tolerance	Identical placebo	Pittsburgh sleep quality index global score (44); adverse effects	RCT; 54
Pinkerton et al. (28)	Hot flashes in menopause	593	54 ± 6.05	0	≥2	Gabapentin: 600 mg/day initially, increased to 1,800 mg/day (600 mg with breakfast and 1,200 mg with the evening meal) from the seventh day	Identical placebo	Mean sleep interference score^c^; adverse effects	RCT; 24
Wallace et al. (34)	Postherpetic neuralgia	400	66.67 ± 12.55	52	≥0.25	Gabapentin: 1,800 mg at night for Group 1 and 600 mg in the morning and 1,200 mg at night for Group 2	Identical placebo	Mean sleep interference score^c^; adverse effects	RCT; 10
Irving et al. (22)	Postherpetic neuralgia	158	69.37 ± 11.59	46.84	≥0.25	Gabapentin: 1,800 mg at night for Group 1 and 600 mg in the morning and 1,200 mg at night for Group 2	Identical placebo	Mean sleep interference score^c^; adverse effects	RCT; 4
Lee et al. (26)	RLS	322	48.95 ± 12.56	58.60	15.56 ± 12.09	Gabapentin enacarbil: 600 mg/day for Group 1 and 1,200 mg/day (once daily at 5:00 p.m. for Group 2)	Identical placebo	Pittsburgh sleep diary, post-sleep questionnaire; adverse effects	RCT; 12
Kushida et al. (23)	RLS	221	51.12 ± 12.80	40.27	14.07 ± 13.78	Gabapentin(XP13512) 1,200 mg once daily at 5:00 p.m.	Identical placebo	Medical outcomes study sleep problems index score (45); post-sleep questionnaire; Pittsburgh Sleep Diary (46); adverse effects	RCT; 12
Kushida et al. (23)	RLS	76	50.1 ± 13.2	42.11	14.30 ± 14.09	Gabapentin(XP13512) 1,800 mg/day during Period 1 followed by placebo during Period 2	Placebo during Period 1, followed by Gabapentin (XP13512) 1,800 mg/day during Period 2	Polysomnography parameters; adverse effects	RCT plus crossover; 4
Walters et al. (35)	RLS	95	50.44 ± 11.17	37.89	16.0 ± 13.11	Gabapentin enacarbil: the dose was 600 mg/day for Group 1 and 1,200 mg/day (once a day at 5:00 p.m.) for Group 2	Identical placebo	Post-sleep questionnaire; adverse effects	RCT; 2
Backonja et al. (16)	Postherpetic neuralgia	102	64.47 ± 12.47	45.54	3.27 ± 4.11	Gabapentin enacarbil: 1,200 mg twice daily	Identical placebo	Mean sleep interference score^c^; adverse effects	RCT; 2
Sang et al. (32)	Postherpetic neuralgia	450	65.61 ± 12.22	37.39	1.68 ± 1.17	Gastroretentive gabapentin: 1,800 mg/day	Identical placebo	Mean sleep interference score^c^; adverse effects	RCT; 11
Sandercock et al. (31)	Diabetic neuralgia	147	58.68 ± 8.24	55.10	10.14 ± 8.72	Gastroretentive gabapentin: 3,000 mg at night for Group 1 and 1,200 mg in the morning and 1,800 mg at night for Group 2	Identical placebo	Mean sleep interference score^c^; adverse effects	RCT; 4
Bogan et al. (17)	RLS	190	51.45 ± 11.90	59.07	14.01 ± 14.13	Gabapentin enacarbil: 1,200 mg once daily	Gabapentin enacarbil at a dose of 600 mg and one tablet of placebo during the first 2 weeks, two placebo tablets from the third week	Post-sleep questionnaire (23); medical Outcomes Study Sleep Scale; kilogram effects	RCT; 12 (excluding the open-label period)
Yurcheshen et al. (37)	Hot flashes in menopause	59	52.85 ± 3.34	0	4.17 ± 3.77	Gabapentin: 300 mg three times daily	Identical placebo	Pittsburgh Sleep Quality Index global score (44); adverse effects	RCT; 12

*RCT, randomized controlled trial; RLS, restless legs syndrome; NR, not reported*.

*^a^Results are shown as the mean ± SD*.

*^b^Results are shown as the median (interquartile ranges)*.

*^c^The range is 0–10, with 0 = no sleep interference and 10 = worst possible sleep interference*.

*^d^Results are shown as the mean (range)*.

*^e^The range is 0–100, with 0 = no sleep interference and 100 = worst possible sleep interference*.

### Quality Assessment

There were seven trials (14, 16, 18, 19, 32, 33, 41) (26.92%) with random sequence generation (14, 16, 18, 19, 22, 32, 33, 41) (30.77%) with allocation concealment, eight trials (14, 16, 18, 19, 22, 32, 33, 41) (30.77%) with blinding of participants, and three trials (16, 32, 41) (11.54%) with blinding of personnel treating the patients and outcome assessors. Except for the 26 trials above that had unclear risks, the trials included in this study had low risks of bias (Figures S1 and S2 in Supplementary Material).

### Efficacy

**Figure 2:**
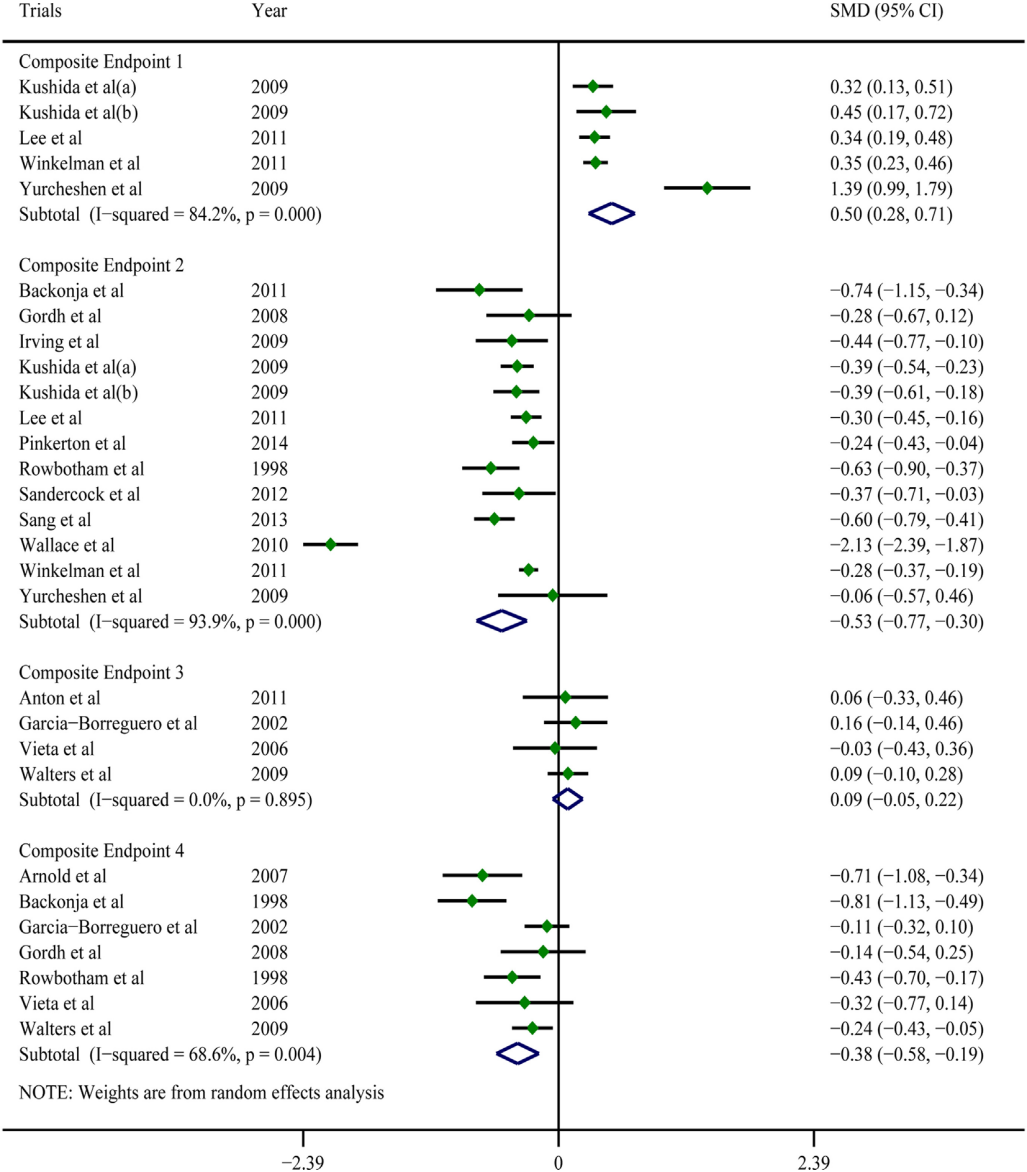
Forest plots of Composite Endpoint 1, Composite Endpoint 2, Composite Endpoint 3, and Composite Endpoint 4. Except for Composite Endpoint 3, the treatment effects of gabapentin were superior to those of the placebo; a random-effects model.

**Figure 3:**
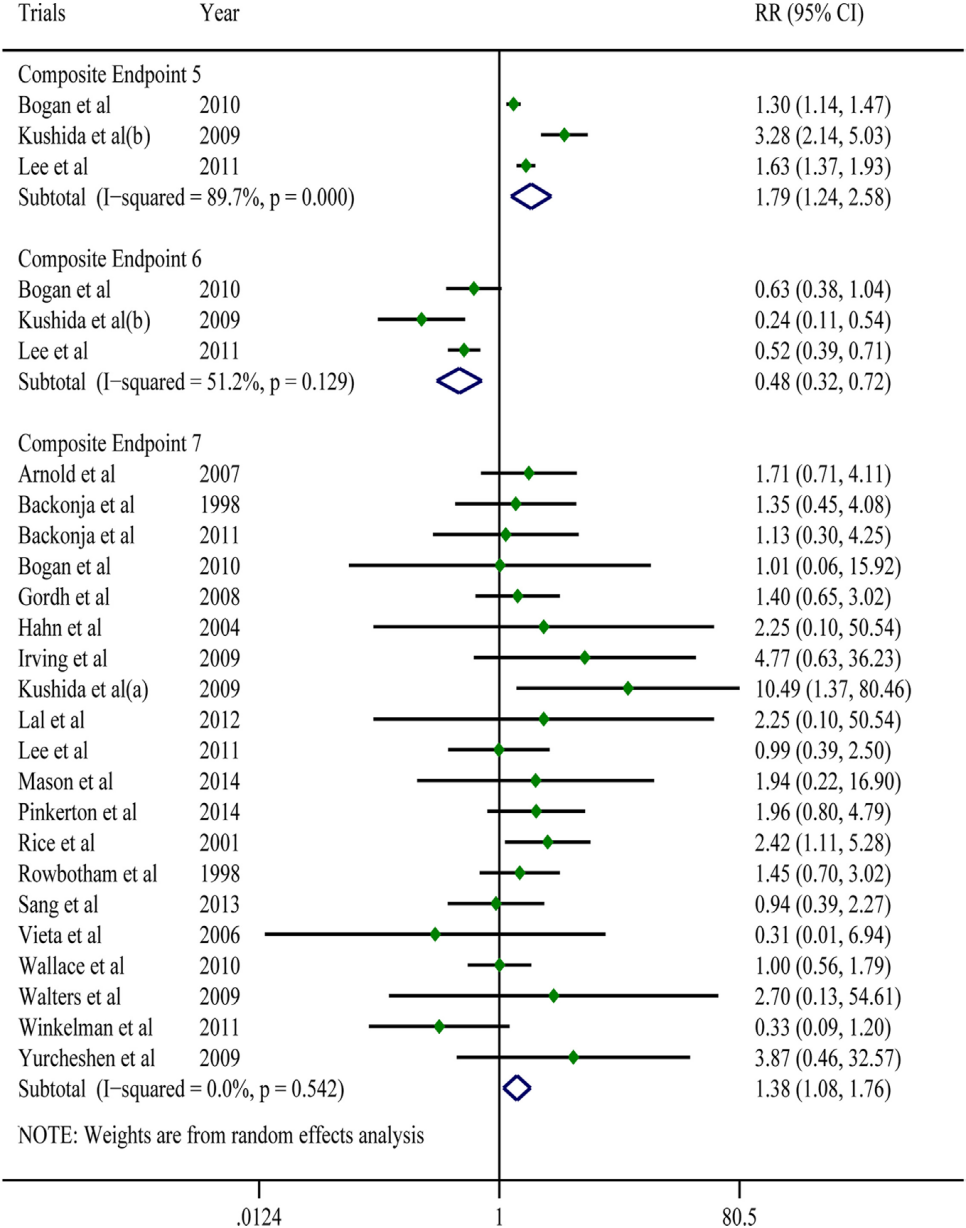
Forest plots of Composite Endpoint 5, Composite Endpoint 6 and Composite Endpoint 7.The treatment effects of gabapentin were superior to those of the placebo; the tolerability of gabapentin was lower than that of the placebo; a random-effects model.

A pooled analysis of eight trials (21, 23, 25–27, 29, 40, 41) demonstrated that other than some indicators in three trials (26, 40, 41), gabapentin showed a treatment efficacy superior to that of the placebos in all trials (Table 2). Regarding multiple subjective and objective sleep indices, the meta-analyses indicated that, except for Composite Endpoint 3 (13, 18, 33, 35) [SMD = 0.09, 95% confidence interval (CI): −0.05–0.22], Composite Endpoint 1 (23, 24, 26, 36, 37), Composite Endpoint 2 (16, 19, 22–24, 26, 28, 30–32, 34, 35, 37), Composite Endpoint 4 (14, 15, 18, 19, 30, 33, 35), Composite Endpoint 5 (17, 26), and Composite Endpoint 6 (17, 26) confirmed that gabapentin’s treatment efficacy was superior to that of the placebos (Figures 2 and 3).

**Table 2:** Efficacy comparison of gabapentin and placebos.

Trials	Endpoints
Kushida et al. (23)	Compared with the placebo group, the gabapentin group showed significant improvement in sleep quality (*P* < 0.001), next-day functioning (*P* < 0.001), number of nighttime awakenings caused by RLS symptoms (*P* = 0.043), and number of hours awake due to RLS symptoms (*P* = 0.019) after 12 weeks of treatment; the gabapentin group had a significantly prolonged total sleep time after 2 weeks of treatment (*P* = 0.003), but there was no statistically significant difference between the two groups after 12 weeks of treatment (*P* = 0.187)
Lee et al. (26)	Compared with the placebo group, the patients who received the treatment at a dose of 600 mg had a significantly shortened average daily wake time after sleep onset at all studied time points (*P* < 0.05) with no increase in their total sleep time (*P* > 0.05)
Hahn et al. (21)	Compared with the placebo group, the gabapentin group showed a significantly improved mean sleep interference score (*P* < 0.05)
Lal et al. (25)	Compared with the placebo group, the gabapentin group showed a significant improvement in all sleep indices (an excellent overall quality of sleep, an excellent ability to function, fewer nights with RLS symptoms, fewer awakenings during the night, 0 or less than 1 h awake per night because of RLS symptoms)
Mason et al. (27)	Compared with the placebo group, the gabapentin group (1,800 mg) had a significantly improved Pittsburgh Sleep Quality Index total score (*P* < 0.001)
Rice et al. (29)	Compared with the placebo group, the gabapentin group (1,800 and 2,400 mg) had a significantly improved mean sleep interference score (*P* < 0.01)
Bone et al. (40)	In terms of the mean sleep interference score, neither the gabapentin group nor the placebo group showed a statistically significant difference (*P* > 0.05)
Brower et al. (41)	Compared with before treatment, the gabapentin group and the placebo group showed improvement in the subjective indices (Sleep Problems Questionnaire, sleep diaries) and the objective indices (polysomnography parameters: sleep onset latency, sleep efficiency, wake time after sleep onset, total sleep time, percentage of sleep spent in Stage 1, percentage of sleep spent in Stage 2, percentage of slow-wave sleep, and percentage of rapid eye movement sleep), but there was no statistically significant difference between the two groups (*P* > 0.05)

### Tolerability

All of the trials reported mild-to-moderate adverse effects. The moderate adverse effects occurred primarily during the dose-increasing phase and significantly decreased in frequency afterward. Drowsiness, dizziness, and weakness were the most frequently reported effects. These discomforts were tolerable for the majority of patients but resulted in drug withdrawal in a portion of patients. A meta-analysis of 20 trials (7, 14–17, 19, 21, 22, 25–28, 32–37) showed that for adverse events that were possibly or probably related to the study drug and could lead to treatment discontinuation and drug withdrawal, the gabapentin group had a 1.45-times higher risk than the placebo group (RR = 1.38; 95% CI: 1.08–1.76; Figure 3); For adverse events that were possibly or probably related to the study drug and could lead to treatment discontinuation and drug withdrawal, the incidences in the gabapentin group and the placebo group were 8.19 and 5.37% (*P* < 0.001), respectively. Sixteen trials (14, 17, 19, 21, 22, 25–29, 31–34, 36) reported serious adverse effects. However, other than one case of headache (34), one case of serious dizziness and drowsiness (21), and one case of vision disturbance (19), no serious adverse effects were associated with the use of gabapentin. No serious adverse events associated with the use of placebos were found.

### GRADE Classification

For the GRADE classifications of evidence quality, the high, moderate, low, and extremely low were 0, 3, 3, and 0, respectively (Table 3).

**Table 3:** Summary of the ratings regarding the quality of evidence.

Outcomes	Illustrative comparative risks^a^ (95% CI)		Relative effect (95% CI)	No of participants (studies)	Quality of the evidence [Grading of Recommendations Assessment, Development and Evaluation (GRADE)]
	Assumed risk	Corresponding risk			
	Placebo	Gabapentin			
Composite Endpoint 1 follow-up: mean 9.67 weeks		The mean Composite Endpoint 1 in the intervention groups was 0.53 SDs higher (0.41–0.66 higher)		2,797 (5 studies)	⊕⊕⊕⊖moderate^b^
Composite Endpoint 2 follow-up: mean 10.28 weeks		The mean Composite Endpoint 2 in the intervention groups was 0.45 SDs lower (0.61–0.3 lower)		5,841 (13 studies)	⊕⊕⊖⊖low^b,c^
Composite Endpoint 4 follow-up: mean 8.09 weeks		The mean Composite Endpoint 4 in the intervention groups was 0.53 SDs lower (0.69–0.36 lower)		1,501 (7 studies)	⊕⊕⊖⊖low^b,c^
Composite Endpoint 5 follow-up: mean 10.97 weeks	Study population 309 per 1,000 Moderate	526 per 1,000 (383–798)	RR 1.7 (1.24–2.58)	2,910 (3 studies)	⊕⊕⊖⊖low^b,c^
Composite Endpoint 6 follow-up: mean 10.97 weeks	Study population 122 per 1,000 Moderate	59 per 1,000 (39–88)	RR 0.48 (0.32–0.72)	2,910 (3 studies)	⊕⊕⊕⊖moderate^c^
Composite Endpoint 7 follow-up: mean 11.60 weeks	Study population 54 per 1,000Moderate	74 per 1,000 (58–94)	RR 1.38 (1.08–1.76)	4,097 (20 studies)	⊕⊕⊕⊖moderate^c^

*^a^The basis for the assumed risk (e.g., the median control group risk across studies) is provided in footnotes. The corresponding risk (and its 95% confidence interval) is based on the assumed risk in the comparison group and the relative effect of the intervention (and its 95% CI)*.

*CI, confidence interval; RR, risk ratio*.

*GRADE Working Group grades of evidence*.

*High quality: further research is very unlikely to change our confidence in the estimate of effect*.

*Moderate quality: further research is likely to have an important impact on our confidence in the estimate of effect and may change the estimate*.

*Low quality: further research is very likely to have an important impact on our confidence in the estimate of effect and is likely to change the estimate*.

*Very low quality: we are very uncertain about the estimate*.

*^b^The differences exist among the trial’s objects*.

*^c^The variation in point estimates among different trials was relatively large, and the heterogeneity test showed results of P < 0.10 and I^2^ > 40%*.

### Sensitivity Analysis

The sensitivity analysis indicated that, for Composite Endpoint 1, the removal of any one trial led to a lower limit of the CI of SMD that was higher than 0; for Composite Endpoint 2 and Composite Endpoint 4, the removal of any one trial led to an upper limit of the CI of SMD that was lower than 0; for Composite Endpoint 5 and Composite Endpoint 7, the removal of any one trial led to a lower limit of the CI of the RR that was higher than 1; for Composite Endpoint 6, the removal of any one trial led to the lower limit of the CI of the RR that was lower than 1 (Figures S3–S8 in Supplementary Material). The above results suggest that the results for these endpoints were robust and had a low sensitivity.

### Publication Bias

The *P* values of all endpoints derived from the Egger test were greater than 0.05, indicating there was no publication bias (Table 4).

**Table 4:** Results of the Egger test.

Results	Composite Endpoint 1	Composite Endpoint 2	Composite Endpoint 4	Composite Endpoint 5	Composite Endpoint 6	Composite Endpoint 7
*P-*value	0.241	0.053	0.063	0.138	0.567	0.336
95% CI	−8.18–3.04	−0.12–14.04	−18.67–0.69	−11.28–23.83	−32.97–29.02	−0.51–1.43

*CI, confidence interval*.

## Discussion

This study revealed that without consideration of the type of sleep outcomes, gabapentin was significantly superior to placebos for the treatment for sleep disorders secondary to RLS, neuropathic pain, alcohol dependence, hot flashes in menopause, fibromyalgia, phantom limb pain, HIV-associated sensory neuropathies, and bipolar disorder. However, with an average dose of approximately 1,800 mg/day, gabapentin had a higher risk of treatment discontinuation and drug withdrawal compared with placebo.

The above conclusion was drawn from an extensive summary of trials involving various primary diseases. Only a small portion of these trials reported the baseline sleep status (14, 18, 21, 26, 27, 36, 37, 41), and none of these trials reported the sleep status prior to the disease. Because it was impossible to distinguish absolutely true, partially true, and false sleep disturbance, we could not exclude the contribution of false sleep disturbance to the final treatment efficacy in patients with medical illness. However, it is worth noting that more than 90% of the patients in these trials had an average disease course of 6.23 (±9.76) years. In terms of the psychological aspects of insomnia, the intention to fall sleep often becomes a driving factor of sleep difficulty (47) and worries about being sleepless often cause early awakening or anxiety (48), particularly among patients who are prone to excessive worry or over thinking. Without timely correction, one episode of sleep difficulty can easily induce a second episode in patients with related psychological traits, and as a result, ongoing sleep difficulties ultimately lead to a chronic sleep disorder. Some researchers believe that the initiating event does not significantly affect the progression of chronic sleep disorders (49) and that chronic sleep disorders are not closely associated with primary disease and thus do not improve with the improvement of the primary disease. In other words, during the chronic course of the abovementioned primary diseases, false sleep disturbance might have transformed into true or partially true sleep disturbance in patients with medical illness for the majority of the sample pool. Thus, we believe the existence of false sleep disturbance in medical illness would not significantly affect the results of the efficacy analysis, and the improvement of sleep disorders can be attributed to the efficacy of gabapentin treatment. The following experimental evidence supports this deduction: gabapentin can shorten sleep latency (36), reduce awakenings (12, 26, 35, 36), reduce fast-wave sleep (23), enhance slow-wave sleep (9–12, 18, 36), prolong the total sleep time (18, 23, 36), increase sleep efficiency (12, 18, 36), and improve the quality of sleep (17, 23, 35, 36). In fact, because of its sedative effect in various diseases, gabapentin has been clinically used as a hypnotic (48). Nevertheless, its efficacy for primary sleep disorders remains to be verified by randomized controlled trials, and the optimal dosage that is effective and tolerable in most patients needs to be identified.

It is necessary to emphasize that despite its insignificant impact on the progression of sleep disorders, the initial sleep difficulty can induce the recurrence of disease (49). In other words, the complete cure of sleep disorders requires a complete removal of the initiating stimulus. Therefore, the use of gabapentin in the abovementioned diseases can “kill two birds with one stone.”

Moreover, it is worth noting that pooled statistics were used with the basic premise of analyzing the efficacy of gabapentin. In this study, we introduced the concept of “composite endpoints” to pool sleep-outcome data that had similar significance and consistent direction. In a broad sense, this research method is in accordance with the basic principle of meta-analysis (39).

### Research Significance

Through a systematic review and meta-analysis, this study for the first time systematically evaluated the clinical value of gabapentin for the treatment of sleep disorders. Used as a starting point, this study could inspire more researchers to conduct in-depth research on this topic.

### Study Limitations

Because of the difficulty of distinguishing false sleep disturbance from true ones in patients with medical illness, we were unable to exclude their contribution to the treatment efficacy. In addition, because of the limitations of the original trials, we were unable to conduct a meta-analysis of individual sleep outcomes and analyses related to treatment dose and timing or patient gender.

## Conclusion

This is the first study to systematically evaluate the clinical value of gabapentin for the treatment of sleep disorders. Regardless the type of sleep outcomes, gabapentin showed stable efficacy in the treatment for sleep disturbance in patients with medical illness with a relatively high risk of treatment discontinuation and drug withdrawal when used at an average dose of approximately 1,800 mg/day. Because the adverse events often occurred during the dose-increasing phase, and the dose was high, reducing the dose-increasing speed and lowering the dosage of gabapentin might reduce the risk. In addition, it would be ideal if our conclusions could be further verified in patients with primary sleep disorders.

## Author Contributions

All authors contributed equally to this work.

## Conflict of Interest Statement

The authors report no potential conflicts of interest with respect to the research, authorship, and/or publication of this article.
