# Supplementary material for: Efficacy and Tolerability of Gabapentin in Adults with Sleep Disturbance in Medical Illness: A Systematic Review and Meta-analysis
Source: Front Neurol. 2017 Jul 14;8:316. doi: 10.3389/fneur.2017.00316 (PMC5510619; doi:10.3389/fneur.2017.00316)
Supplement: Supplementary file 1 [file Data_Sheet_1.DOC]

**Supplementary Materials**

**Tables: 9**

Table 1 Composite Endpoint 1

| Trials | Gabapentin | | | Placebo | | | Sleep outcome |
| --- | --- | --- | --- | --- | --- | --- | --- |
|  | Sample size | Mean | Standard deviation | Sample size | Mean | Standard deviation |  |
| Kushida et al (a), 2009^20^ | 113 | 0.8 | 1.24 | 108 | 0.4 | 1.38 | MOS: sleep quantity |
| Kushida et al (a), 2009^20^ | 113 | 27.7 | 29.89 | 108 | 13.4 | 27.42 | MOS: sleep adequacy |
| Kushida et al (b), 2009^21^ | 34 | 22.4 | 40.8 | 34 | 1.4 | 34.3 | Stage N3/N4 sleep time |
| Kushida et al (b), 2009^21^ | 34 | 24.5 | 53.1 | 34 | -0.6 | 54.8 | Total sleep time |
| Kushida et al (b), 2009^21^ | 34 | 5.1 | 11.1 | 34 | -0.1 | 11.4 | Sleep efficiency |
| Lee et al, 2011^22^ | 111 | 1 | 1.32 | 96 | 0.6 | 1.36 | Total sleep time |
| Lee et al, 2011^22^ | 226 | 0.7 | 1.47 | 96 | 0.3 | 1.19 | MOS: sleep quantity |
| Lee et al, 2011^22^ | 226 | 28.41 | 29.85 | 96 | 13.6 | 24.59 | MOS: sleep adequacy |
| Winkelman et al, 2011^30^ | 121 | 15.02 | 26.08 | 123 | 2.89 | 26.07 | PSG: stage N3 sleep time |
| Winkelman et al, 2011^30^ | 121 | 7.57 | 12.87 | 123 | 0.76 | 12.88 | PSG: sleep efficiency |
| Winkelman et al, 2011^30^ | 121 | 36.32 | 61.77 | 123 | 3.62 | 61.8 | PSG: total sleep time |
| Winkelman et al, 2011^30^ | 109 | 1.95 | 1.51 | 116 | 0.85 | 1.52 | SPSD: sleep quality |
| Winkelman et al, 2011^30^ | 109 | 1.68 | 1.52 | 116 | 0.83 | 1.53 | SPSD: feeling rested upon awakening |
| Yurcheshen et al, 2009^31^ | 30 | 1.27 | 0.55 | 29 | 0.28 | 0.54 | PSQI: sleep quality |
| Yurcheshen et al, 2009^31^ | 30 | 0.94 | 0.55 | 29 | 0.39 | 0.54 | PSQI: sleep efficiency |

PSG=Polysomnography parameters; SPSD=Subjective Post-Sleep Diary; MOS=Medical Outcomes Study; PSQI=Pittsburgh Sleep Quality Index

Table 2 Composite Endpoint 2

| Trials | Gabapentin | | | Placebo | | | Sleep outcome |
| --- | --- | --- | --- | --- | --- | --- | --- |
|  | Sample size | Mean | Standard deviation | Sample size | Mean | Standard deviation |  |
| Backonja et al, 2011^15^ | 47 | -2.2 | 1.76 | 54 | -0.9 | 1.75 | Mean sleep interference score |
| Gordh et al, 2008^18^ | 48 | -10.2 | 15.6 | 50 | -6.3 | 12.5 | Mean sleep interference score |
| Irving et al, 2009^19^ | 107 | -2.11 | 2.19 | 51 | -1.16 | 2.14 | Mean sleep interference score |
| Kushida et al (a), 2009^20^ | 113 | -17.4 | 19.39 | 108 | -9.6 | 17.18 | Daytime somnolence |
| Kushida et al (a), 2009^20^ | 113 | -29.1 | 23.84 | 108 | -15.5 | 21.79 | Sleep disturbance |
| Kushida et al (a), 2009^20^ | 113 | -17.6 | 29.28 | 108 | -11.8 | 24.91 | Wake time after sleep onset |
| Kushida et al (b), 2009^21^ | 34 | -9.8 | 23.9 | 34 | 0.4 | 23.2 | Stage N1 sleep |
| Kushida et al (b), 2009^21^ | 34 | -21.5 | 50.2 | 34 | 6.7 | 38.2 | Wake time after persistent sleep onset |
| Kushida et al (b), 2009^21^ | 34 | -14.1 | 38.9 | 34 | 11.5 | 33.8 | Wake time during sleep |
| Kushida et al (b), 2009^21^ | 34 | -2.1 | 4.0 | 34 | 0.4 | 3.8 | Number of awakenings |
| Kushida et al (b), 2009^21^ | 34 | -4.4 | 27.2 | 34 | -7.4 | 40.5 | Latency to persistent sleep |
| Rowbotham et al, 1998^24^ | 109 | -1.9 | 2.5 | 116 | -0.5 | 1.9 | Mean sleep interference score |
| Lee et al, 2011^22^ | 226 | -12.89 | 20.18 | 96 | -9.7 | 20.29 | MOS: daytime somnolence |
| Lee et al, 2011^22^ | 111 | -16.72 | 27.66 | 96 | -12.5 | 31.01 | Wake time after sleep onset |
| Lee et al, 2011^22^ | 226 | -30.09 | 24.32 | 96 | -17 | 20.4 | MOS: sleep disturbance |
| Pinkerton et al, 2014^23^ | 206 | -3.2 | 1.44 | 191 | -2.2 | 1.38 | Mean sleep interference score |
| Sandercock et al, 2012^25^ | 96 | -2.74 | 2.57 | 51 | -1.79 | 2.56 | Mean sleep interference score |
| Sang et al, 2013^26^ | 220 | -2.7 | 1.48 | 230 | -1.8 | 1.52 | Mean sleep interference score |
| Wallace et al, 2010^28^ | 269 | -2.24 | 0.32 | 131 | -1.63 | 0.2 | Mean sleep interference score |
| Winkelman et al, 2011^30^ | 121 | -32.02 | 55.22 | 123 | -6.02 | 55.3 | PSG: wake time during sleep |
| Winkelman et al, 2011^30^ | 121 | -6.88 | 40.96 | 123 | -0.23 | 41.03 | PSG: latency to persistent sleep |
| Winkelman et al, 2011^30^ | 121 | -3.04 | 4.55 | 123 | -0.56 | 4.56 | PSG: number of awakenings |
| Winkelman et al, 2011^30^ | 121 | -32.24 | 55.02 | 123 | -3.76 | 55.05 | PSG: wake time after sleep onset |
| Winkelman et al, 2011^30^ | 121 | -4.02 | 29.87 | 123 | 0.21 | 29.91 | PSG: sleep onset latency |
| Winkelman et al, 2011^30^ | 121 | -4.59 | 9.89 | 123 | -1.52 | 9.89 | PSG: PLMs associated with arousal per hour of sleep |
| Winkelman et al, 2011^30^ | 121 | -0.22 | 0.53 | 123 | -0.08 | 0.52 | PSG: PLMs associated with awakening per hour of sleep |
| Winkelman et al, 2011^30^ | 121 | -12.66 | 25.32 | 123 | -4.61 | 25.36 | PSG: periodic limb movement index |
| Yurcheshen et al, 2009^31^ | 30 | -0.6 | 0.55 | 29 | -0.57 | 0.54 | PSQI: daytime dysfunction |

PSG=Polysomnography parameters; PLMs=Periodic Limb Movements; MOS=Medical Outcomes Study; PSQI=Pittsburgh Sleep Quality Index

Table 3 Composite Endpoint 3

| Trials | Gabapentin | | | Placebo | | | Sleep outcome |
| --- | --- | --- | --- | --- | --- | --- | --- |
|  | Sample size | Mean | Standard deviation | Sample size | Mean | Standard deviation |  |
| Anton et al, 2011^12^ | 48 | 10.1 | 6 | 49 | 9.7 | 6.6 | Insomnia Sleep Index |
| Garcia-Borreguero et al, 2002^17^ | 22 | 6.0 | 0.47 | 22 | 5.5 | 0.47 | Total sleep time |
| Garcia-Borreguero et al, 2002^17^ | 22 | 84.7 | 7.04 | 22 | 74.9 | 7.04 | Sleep efficiency |
| Garcia-Borreguero et al, 2002^17^ | 22 | 104.5 | 35.18 | 22 | 82.2 | 35.18 | Slow wave sleep |
| Garcia-Borreguero et al, 2002^17^ | 22 | 52.7 | 20.17 | 22 | 60 | 20.17 | Stage REM |
| Vieta et al, 2006^27^ | 13 | 1.0 | 0.9 | 12 | 1.1 | 0.9 | PSQI: sleep quality |
| Vieta et al, 2006^27^ | 13 | 0.5 | 0.7 | 12 | 0.4 | 0.9 | PSQI: sleep duration |
| Vieta et al, 2006^27^ | 13 | 1.0 | 0.4 | 12 | 1.0 | 0.3 | PSQI: sleep duration |
| Vieta et al, 2006^27^ | 13 | 0.5 | 0.5 | 12 | 0.6 | 0.9 | PSQI: habitual sleep efficiency |
| Walters et al, 2009^29^ | 61 | 14.25 | 47.93 | 33 | 9 | 27.3 | Overall quality of sleep in the past 7 days: good |
| Walters et al, 2009^29^ | 61 | 23.57 | 76.43 | 33 | 27 | 81.8 | Ability to function in the past 7 days: good |
| Walters et al, 2009^29^ | 61 | 7.67 | 26.70 | 33 | 1 | 3.0 | Number of nights with RLS symptoms: 0 |
| Walters et al, 2009^29^ | 61 | 16.77 | 56.09 | 33 | 11 | 33.3 | Number of nighttime awakenings due to RLS symptoms: 0 |
| Walters et al, 2009^29^ | 61 | 7.67 | 26.70 | 33 | 1 | 3.0 | Number of hours awake per night due to RLS symptoms: 0 |

RLS=Restless legs syndrome; PSQI=Pittsburgh Sleep Quality Index; REM=Rapid eye movement

Table 4 Composite Endpoint 4

| Trials | Gabapentin | | | Placebo | | | Sleep outcome |
| --- | --- | --- | --- | --- | --- | --- | --- |
|  | Sample size | Mean | Standard deviation | Sample size | Mean | Standard deviation |  |
| Arnold et al, 2007^13^ | 57 | 33.4 | 19.5 | 62 | 47.8 | 20.9 | Medical Outcomes Study Sleep Problems Index score |
| Backonja et al, 1998^14^ | 82 | 2.3 | 1.88 | 80 | 3.8 | 1.83 | Mean sleep interference score |
| Garcia-Borreguero et al, 2002^17^ | 22 | 6.4 | 1.88 | 22 | 9.3 | 1.88 | Pittsburgh Sleep Quality Index |
| Garcia-Borreguero et al, 2002^17^ | 22 | 11.1 | 15.48 | 22 | 20.8 | 15.48 | Periodic leg movements in sleep |
| Garcia-Borreguero et al, 2002^17^ | 22 | 15.9 | 17.82 | 22 | 22.0 | 17.82 | Sleep latency |
| Garcia-Borreguero et al, 2002^17^ | 22 | 103.1 | 32.36 | 22 | 108.9 | 32.36 | Latency to REM sleep |
| Garcia-Borreguero et al, 2002^17^ | 22 | 57.3 | 32.36 | 22 | 80.1 | 32.36 | Sleep latency: stage N1 sleep |
| Garcia-Borreguero et al, 2002^17^ | 22 | 207 | 40.34 | 22 | 216 | 40.34 | Sleep latency: stage N2 sleep |
| Garcia-Borreguero et al, 2002^17^ | 22 | 4.3 | 6.10 | 22 | 5.4 | 6.10 | PLM-arousal index, per hour |
| Garcia-Borreguero et al, 2002^17^ | 22 | 20.3 | 11.73 | 22 | 23.7 | 11.73 | Arousal index, per hour |
| Vieta et al, 2006^27^ | 13 | 1.3 | 1.1 | 12 | 1.7 | 1.0 | PSQI: sleep latency |
| Vieta et al, 2006^27^ | 13 | 0.9 | 1.5 | 12 | 1.5 | 1.3 | PSQI: use of sleep medication |
| Vieta et al, 2006^27^ | 13 | 1.0 | 1.0 | 12 | 1.1 | 1.0 | PSQI: daytime dysfunction |
| Gordh et al, 2008^18^ | 48 | 28 | 26.1 | 50 | 31.4 | 20.9 | Mean sleep interference score |
| Rowbotham et al, 1998^24^ | 109 | 2.4 | 2.5 | 116 | 3.6 | 3.0 | Average daily sleep rating scores |
| Walters et al, 2009^29^ | 61 | 1.43 | 7.20 | 33 | 4.0 | 12.1 | Overall quality of sleep in the past 7 days: poor |
| Walters et al, 2009^29^ | 61 | 0.52 | 2.28 | 33 | 0 | 0 | Ability to function in the past 7 days: poor |
| Walters et al, 2009^29^ | 61 | 2.95 | 10.51 | 33 | 6.0 | 18.2 | Number of nights with RLS symptoms: 7 |
| Walters et al, 2009^29^ | 61 | 0.48 | 2.38 | 33 | 3.0 | 9.1 | Number of nighttime awakenings due to RLS symptoms: ≥5 |
| Walters et al, 2009^29^ | 61 | 0.48 | 2.38 | 33 | 3.0 | 9.1 | Number of hours awake per night due to RLS symptoms: ≥3 |

RLS=Restless legs syndrome; PSQI=Pittsburgh Sleep Quality Index; REM=Rapid eye movement

Table 5 Composite Endpoint 5 (Excellent, 0 or Good)

| Trials | Gabapentin | | | Placebo | | | Sleep outcome |
| --- | --- | --- | --- | --- | --- | --- | --- |
|  | Sample size | G1 | G2 | Sample size | P1 | P2 |  |
| Bogan et al, 2010^16^ | 96 | 38 | 58 | 97 | 29 | 68 | Overall quality of sleep in past week: excellent |
| Bogan et al, 2010^16^ | 96 | 53 | 43 | 97 | 44 | 53 | Ability to function in past week: excellent |
| Bogan et al, 2010^16^ | 96 | 41 | 55 | 97 | 30 | 67 | Number of nights with RLS symptoms: 0 |
| Bogan et al, 2010^16^ | 96 | 68 | 28 | 97 | 53 | 44 | Number of awakenings during the night due to RLS symptoms: 0 |
| Bogan et al, 2010^16^ | 96 | 68 | 28 | 97 | 53 | 44 | Number of hours awake per night due to RLS symptoms: 0 |
| Kushida et al (b), 2009^21^ | 34 | 17 | 17 | 34 | 3 | 31 | Overall quality of sleep: good |
| Kushida et al (b), 2009^21^ | 34 | 25 | 9 | 34 | 14 | 20 | Ability to function: good |
| Kushida et al (b), 2009^21^ | 34 | 7 | 27 | 34 | 1 | 33 | Number of nights with RLS symptoms: 0 |
| Kushida et al (b), 2009^21^ | 34 | 16 | 18 | 34 | 3 | 31 | Number of nighttime awakenings due to RLS symptoms: 0 |
| Kushida et al (b), 2009^21^ | 34 | 8 | 28 | 34 | 1 | 33 | Number of hours awake per night due to RLS symptoms: 0 |
| Lee et al, 2011^22^ | 225 | 54 | 171 | 96 | 14 | 82 | Overall quality of sleep: excellent |
| Lee et al, 2011^22^ | 225 | 84 | 141 | 96 | 23 | 73 | Ability to function: excellent |
| Lee et al, 2011^22^ | 225 | 67 | 158 | 96 | 13 | 86 | Number of nights with RLS symptoms: 0 |
| Lee et al, 2011^22^ | 225 | 125 | 100 | 96 | 35 | 61 | Number of nighttime awakenings due to RLS symptoms: 0 |
| Lee et al, 2011^22^ | 225 | 125 | 100 | 96 | 35 | 61 | Number of hours awake per night due to RLS symptoms: 0 |

RLS=Restless legs syndrome; G1 and P1 are the numbers of patients who showed the specified sleep outcome, and G2 and P2 are the numbers of the patients who did not show the specified sleep outcome

Table 6 Composite Endpoint 6 (Poor, ≥3, ≥5 or 7)

| Trials | Gabapentin | | | Placebo | | | Sleep outcome |
| --- | --- | --- | --- | --- | --- | --- | --- |
|  | Sample size | G1 | G2 | Sample size | P1 | P2 |  |
| Kushida et al (b), 2009^21^ | 34 | 1 | 33 | 34 | 13 | 21 | Overall quality of sleep: poor |
| Kushida et al (b), 2009^21^ | 34 | 1 | 33 | 34 | 2 | 32 | Ability to function: poor |
| Kushida et al (b), 2009^21^ | 34 | 5 | 29 | 34 | 10 | 24 | Number of nights with RLS symptoms: 7 |
| Kushida et al (b), 2009^21^ | 34 | 0 | 34 | 34 | 2 | 32 | Number of nighttime awakenings due to RLS symptoms: ≥5 |
| Kushida et al (b), 2009^21^ | 34 | 0 | 34 | 34 | 2 | 32 | Number of hours awake per night due to RLS symptoms: ≥3 |
| Lee et al, 2011^22^ | 225 | 34 | 191 | 96 | 29 | 67 | Overall quality of sleep in the past week: poor |
| Lee et al, 2011^22^ | 225 | 6 | 219 | 96 | 5 | 91 | Ability to function in the past week: poor |
| Lee et al, 2011^22^ | 225 | 34 | 191 | 96 | 23 | 73 | Number of nights with RLS symptoms: 7 |
| Lee et al, 2011^22^ | 225 | 3 | 122 | 96 | 7 | 89 | Number of nighttime awakenings due to RLS symptoms: ≥5 |
| Lee et al, 2011^22^ | 225 | 5 | 220 | 96 | 9 | 86 | Number of hours awake per night due to RLS symptoms: ≥3 |
| Bogan et al, 2010^16^ | 96 | 12 | 84 | 97 | 17 | 80 | Overall quality of sleep in the past week: poor |
| Bogan et al, 2010^16^ | 96 | 3 | 93 | 97 | 1 | 96 | Ability to function in the past week: poor |
| Bogan et al, 2010^16^ | 96 | 7 | 89 | 97 | 14 | 83 | Number of nights with RLS symptoms: 7 |
| Bogan et al, 2010^16^ | 96 | 1 | 95 | 97 | 2 | 95 | Number of nighttime awakenings due to RLS symptoms: ≥5 |
| Bogan et al, 2010^16^ | 96 | 0 | 96 | 97 | 3 | 94 | Number of hours awake per night due to RLS symptoms: ≥3 |

RLS=Restless legs syndrome; G1 and P1 are the numbers of the patients who showed the specified sleep outcome, and G2 and P2 are the numbers of the patients who did not show the specified sleep outcome

Table 7 Treatment discontinuation or drug withdrawal

| Trials | Gabapentin | | | Placebo | | |
| --- | --- | --- | --- | --- | --- | --- |
|  | Sample size | G1 | G2 | Sample size | P1 | P2 |
| Mason et al, 2014^34^ | 101 | 4 | 97 | 49 | 1 | 48 |
| Lal et al, 2012^33^ | 15 | 1 | 14 | 11 | 0 | 11 |
| Rice et al, 2001^35^ | 223 | 34 | 189 | 111 | 7 | 104 |
| Arnold et al, 2007^13^ | 75 | 12 | 63 | 75 | 7 | 68 |
| Backonja et al, 1998^14^ | 84 | 7 | 77 | 81 | 5 | 76 |
| Backonja et al, 2011^15^ | 48 | 4 | 44 | 54 | 4 | 50 |
| Bogan et al, 2010^16^ | 96 | 1 | 95 | 97 | 1 | 96 |
| Gordh et al, 2008^18^ | 61 | 13 | 48 | 59 | 9 | 50 |
| Irving et al, 2009^19^ | 107 | 10 | 97 | 51 | 1 | 50 |
| Kushida et al (a), 2009^20^ | 113 | 10 | 93 | 108 | 1 | 107 |
| Lee et al, 2011^22^ | 226 | 14 | 212 | 96 | 6 | 90 |
| Pinkerton et al, 2014^23^ | 300 | 14 | 286 | 295 | 7 | 287 |
| Rowbotham et al, 1998^24^ | 109 | 15 | 94 | 116 | 11 | 105 |
| Sang et al, 2013^26^ | 221 | 9 | 212 | 231 | 10 | 221 |
| Vieta et al, 2006^27^ | 13 | 0 | 13 | 12 | 1 | 11 |
| Wallace et al, 2010^28^ | 274 | 31 | 243 | 133 | 15 | 118 |
| Walters et al, 2009^29^ | 62 | 2 | 60 | 33 | 0 | 33 |
| Winkelman et al, 2011^30^ | 136 | 3 | 133 | 136 | 9 | 127 |
| Yurcheshen et al, 2009^31^ | 30 | 4 | 26 | 29 | 1 | 28 |
| Hahn et al, 2004^32^ | 15 | 1 | 14 | 11 | 0 | 11 |

G1 and P1 are the numbers of the patients who showed the specified sleep outcome, and G2 and P2 are the numbers of the patients who did not show the specified sleep outcome.
