# Supplementary material for: Efficacy and Tolerability of Gabapentin in Adults with Sleep Disturbance in Medical Illness: A Systematic Review and Meta-analysis
Source: Front Neurol. 2017 Jul 14;8:316. doi: 10.3389/fneur.2017.00316 (PMC5510619; doi:10.3389/fneur.2017.00316)
Supplement: Supplementary file 2 [file Data_Sheet_2.DOC]

**Appendix Materials**

**Search Terms**

The search term was (Gabapentin OR Gabapentin enacarbil OR XP13512) AND (Insomnia OR Sleeplessness OR Sleep Initiation and Maintenance Disorders OR Sleep OR Sleep Deprivation OR Sleep Disorders OR Sleep Stages OR Sleep Disorders, Circadian Rhythm OR REM Sleep Behavior Disorder OR Sleep Disorders, Intrinsic OR Disorders of Initiating and Maintaining Sleep OR Insufficient Sleep Syndrome OR REM Sleep Deprivation OR Sleep Fragmentation OR Circadian Rhythm Sleep Disorders OR Shift-Work Sleep Disorder OR Sleep-Wake Cycle Disorders OR Rapid Eye Movement Sleep Behavior Disorder) AND (Clinical trial).

**Appendix Figures: 10**

Appendix Figure 1 Risk of bias graph for methodological quality

The results of the risk assessment of bias for all of the included trials are expressed as percentages.

Appendix Figure 2 Risk of bias summary for methodological quality

The result of the risk assessment of bias for each included trial is presented separately.

Appendix Figure 3 Sensitivity analysis of Composite Endpoint 1

After the removal of any one comparison, the lower limit of the CI of SMD was higher than zero.

Appendix Figure 4 Sensitivity analysis of Composite Endpoint 2

After the removal of any one comparison, the upper limit of the CI of SMD was lower than zero.

Appendix Figure 5 Sensitivity analysis of Composite Endpoint 4

After the removal of any one comparison, the upper limit of the CI of SMD was lower than zero.

Appendix Figure 6 Sensitivity analysis of Composite Endpoint 5

After the removal of any one comparison, the lower limit of the CI of RR was higher than 1.

Appendix Figure 7 Sensitivity analysis of Composite Endpoint 6

After the removal of any one comparison, the upper limit of the CI of RR was lower than 1.

Appendix Figure 8 Sensitivity analysis of Composite Endpoint 7

After the removal of any one comparison, the lower limit of the CI of RR was higher than 1
