# Supplementary figures and images for: Efficacy and Tolerability of Gabapentin in Adults with Sleep Disturbance in Medical Illness: A Systematic Review and Meta-analysis
Source: Front Neurol. 2017 Jul 14;8:316. doi: 10.3389/fneur.2017.00316 (PMC5510619; doi:10.3389/fneur.2017.00316)

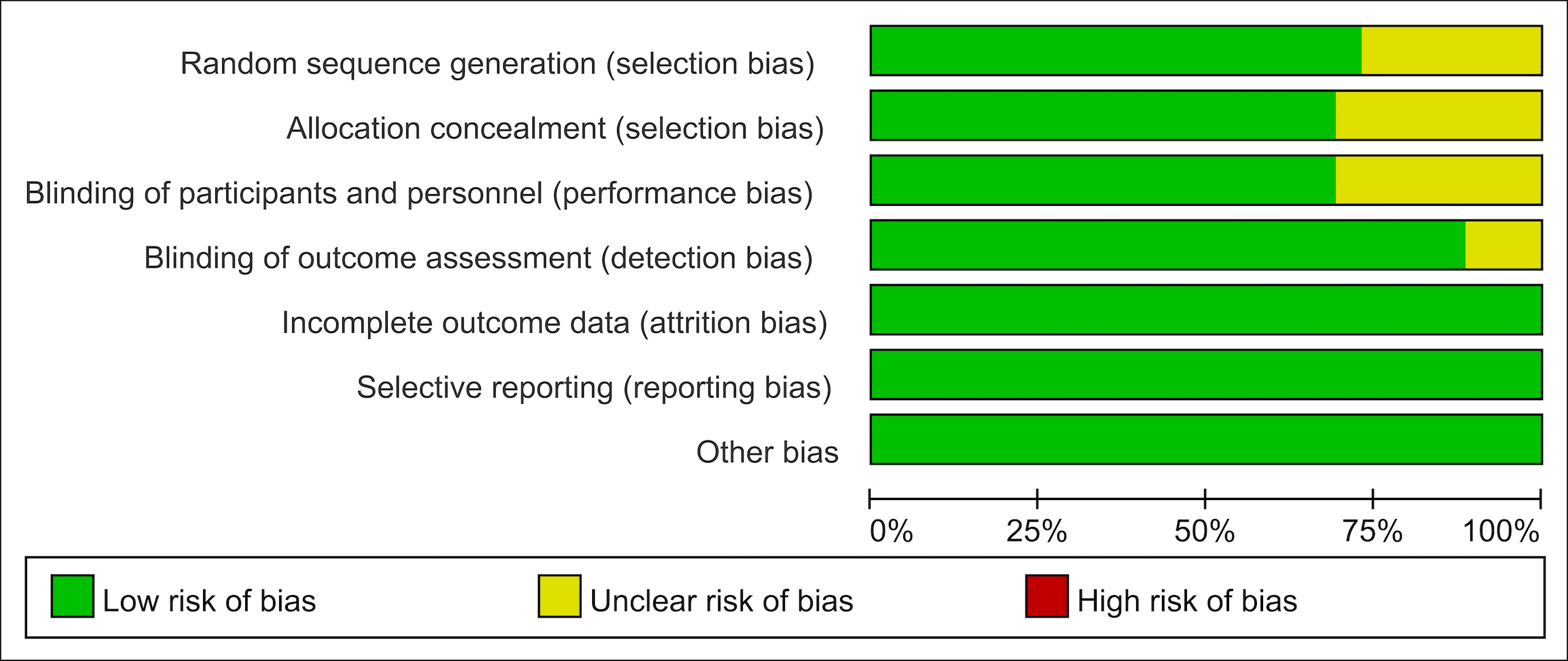

Supplement: Supplementary file 3 [file Image_1.TIF]

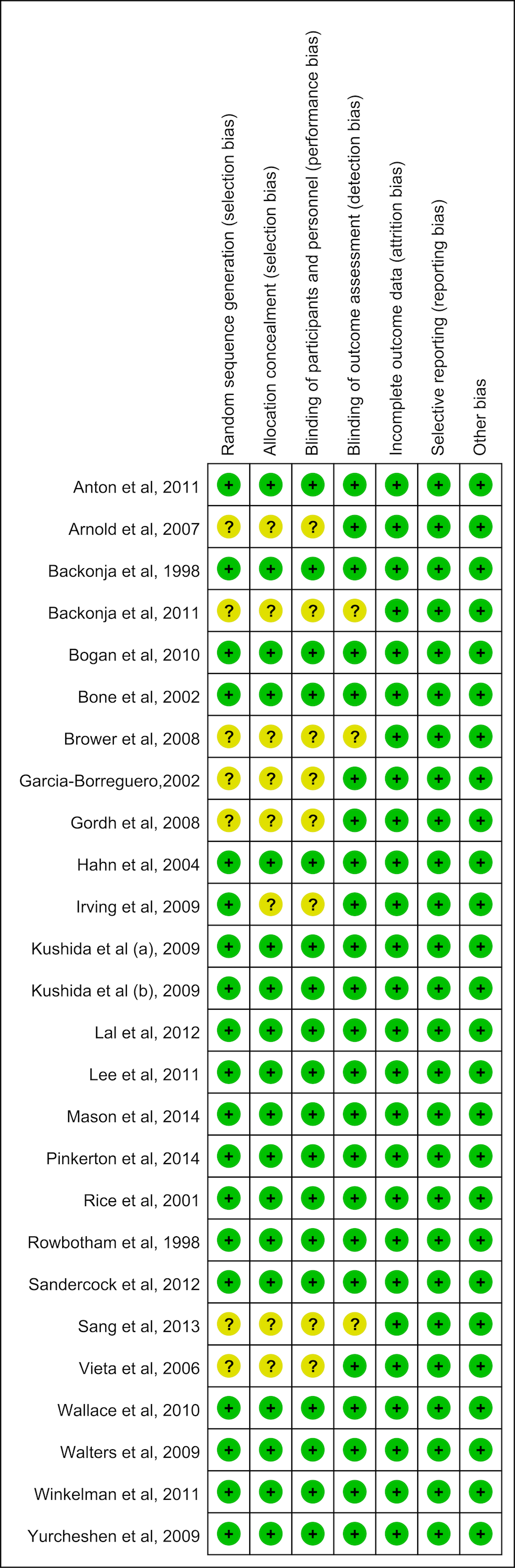

Supplement: Supplementary file 4 [file Image_2.TIF]

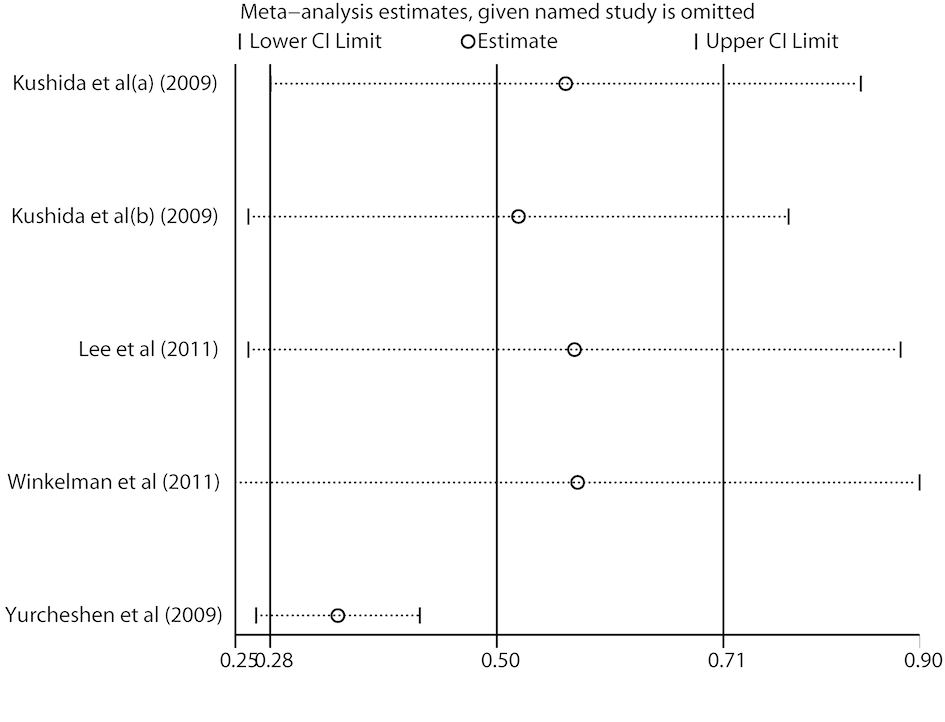

Supplement: Supplementary file 5 [file Image_3.TIF]

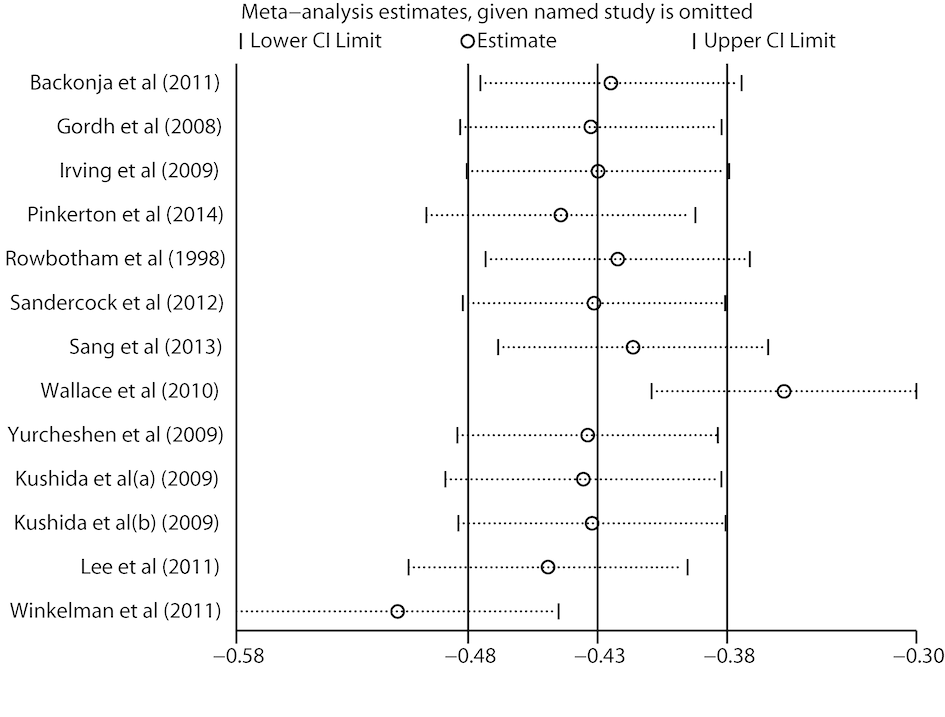

Supplement: Supplementary file 6 [file Image_4.TIF]

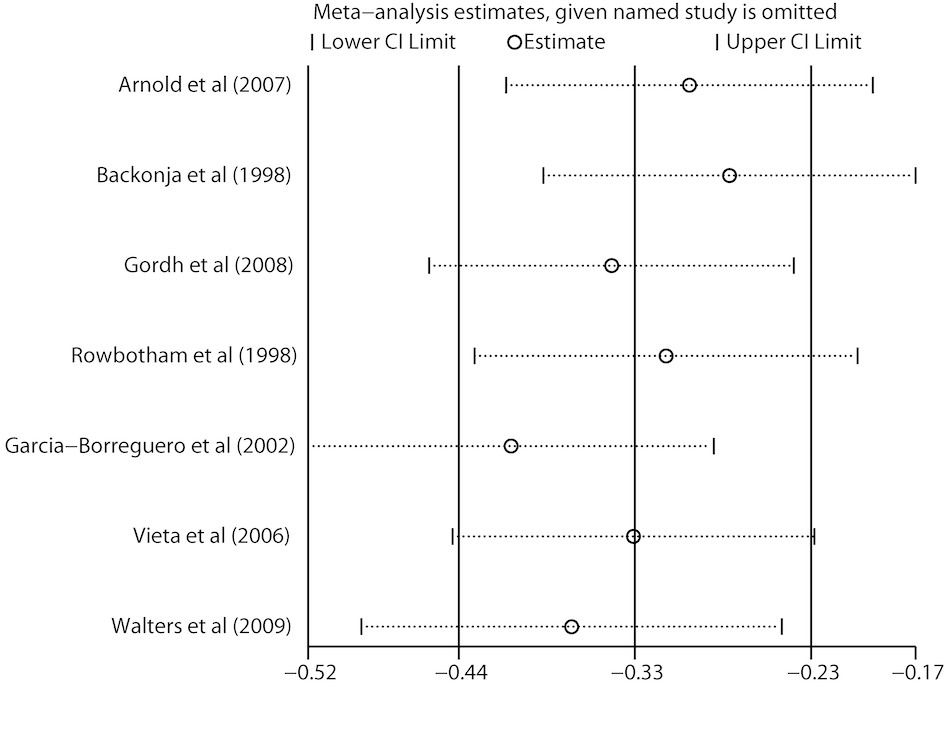

Supplement: Supplementary file 7 [file Image_5.TIF]

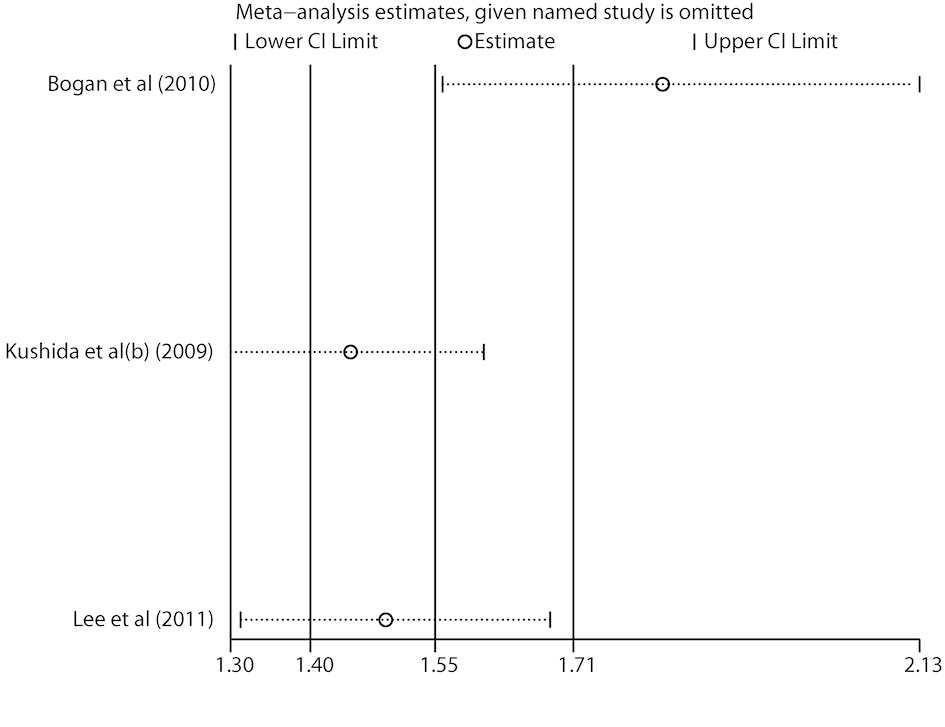

Supplement: Supplementary file 8 [file Image_6.TIF]

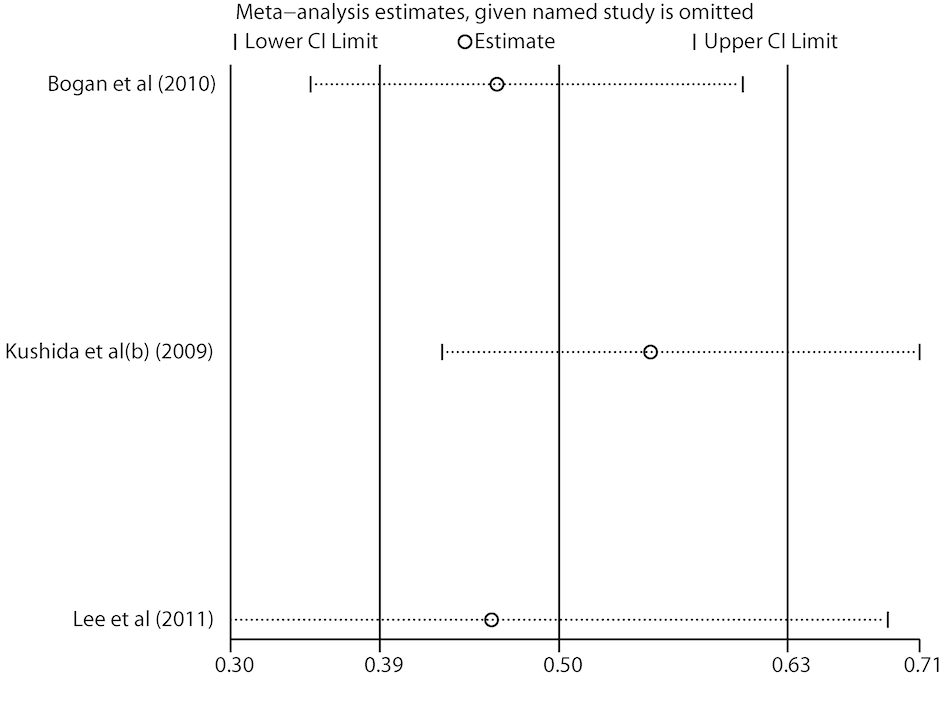

Supplement: Supplementary file 9 [file Image_7.TIF]

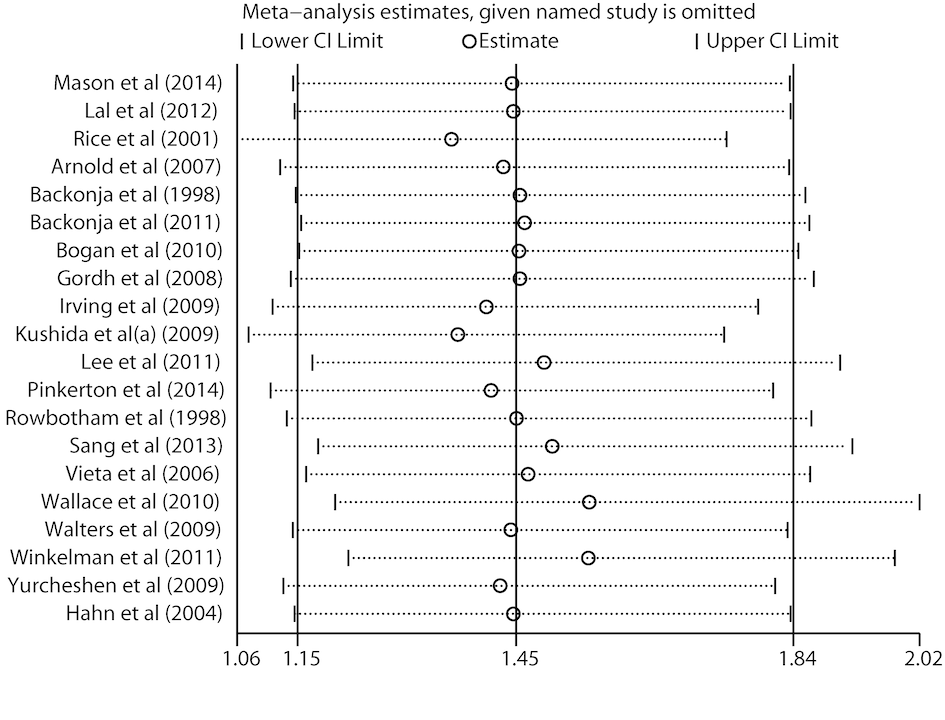

Supplement: Supplementary file 10 [file Image_8.TIF]
